# Motor Neuron Diseases in Sub-Saharan Africa: The Need for More Population-Based Studies

**DOI:** 10.1155/2015/298409

**Published:** 2015-08-12

**Authors:** Emmanuel Quansah, Thomas K. Karikari

**Affiliations:** ^1^Pharmacology, Faculty of Health and Life Sciences, De Montfort University, Leicester LE1 9BH, UK; ^2^Department of Molecular Biology and Biotechnology, School of Biological Science, University of Cape Coast, Cape Coast, Ghana; ^3^Department of Science Laboratory Technology, School of Applied Science and Technology, Wa Polytechnic, Wa, Ghana

## Abstract

Motor neuron diseases (MNDs) are devastating neurological diseases that are characterised by gradual degeneration and death of motor neurons. Major types of MNDs include amyotrophic lateral sclerosis (ALS) and spinal muscular atrophy (SMA). These diseases are incurable, with limited disease-modifying treatment options. In order to improve MND-based biomedical research, drug development, and clinical care, population-based studies will be important. These studies, especially among less-studied populations, might identify novel factors controlling disease susceptibility and resistance. To evaluate progress in MND research in Africa, we examined the published literature on MNDs in Sub-Saharan Africa to identify disease prevalence, genetic factors, and other risk factors. Our findings indicate that the amount of research evidence on MNDs in Sub-Saharan Africa is scanty; molecular and genetics-based studies are particularly lacking. While only a few genetic studies were identified, these studies strongly suggest that there appear to be population-specific causes of MNDs among Africans. MND genetic underpinnings vary among different African populations and also between African and non-African populations. Further studies, especially molecular, genetic and genomic studies, will be required to advance our understanding of MND biology among African populations. Insights from these studies would help to improve the timeliness and accuracy of clinical diagnosis and treatment.

## 1. Introduction

Motor neuron diseases (MNDs) are a group of neurological disorders in which there is a selective loss of function of motor neurons [1]. These diseases are characterised by a progressive loss of motor neurons in the cerebral cortex, motor nuclei of the brain stem, and the anterior horn cells of the spinal cord [1]. The major disease types include amyotrophic lateral sclerosis (ALS), spinal muscular atrophy (SMA), progressive muscular atrophy (PMA), primary lateral sclerosis, and progressive bulbar palsy [2]. These diseases are incurable, with limited disease-modifying treatment options [2, 3]. MNDs share some similarities with other neurological diseases (such as neurodegenerative diseases including Alzheimer's and Parkinson's diseases) particularly in terms of the common genetic defects and the economic, social, and health burdens they present [4, 5].

The mechanistic details underlying the progressive loss of motor neurons in MNDs remain poorly understood [6]. However, an interplay of genetic, age-related, environmental, and developmental factors has been suggested to contribute to disease progression [7]. While some MNDs are genetically inherited (familial), others are sporadic [8, 9]. In ALS, for example, there are both familial and sporadic forms. Different genetic factors such as mutations in the* C9orf72* and superoxide dismutase (*SOD1*) genes have been linked to familial ALS [10]. Furthermore, SMA is an autosomal recessive disorder with onset in infancy (Werdnig-Hoffman disease) or childhood through to adulthood (Kugelberg-Welander syndrome) [11]. Mutations in the Survival Motor Neuron 1 gene (*SMN1*; which is located on chromosome 5q13) have been linked with SMA [12]. Reduced* SMN1* gene expression leads to degeneration of lower motor neurons, resulting in symmetrical muscle weakness and wasting [12].* SMN1* is reported to be homozygously deleted in approximately 95% of SMA patients worldwide [12]. However, studies conducted in South Africa have reported lower homozygous deletions of the* SMN1* gene in patients of black African ancestry (51%–65%) compared to those of white ancestry (90–95%) [13, 14]. It is believed that MNDs have relatively higher prevalence rates among natives of North America, Europe, and Asia, compared to those in Sub-Saharan Africa (SSA). However, the less-studied nature of African populations might mean that the true epidemiological data among these populations are yet to be known.

To evaluate progress in MND research in Africa and to depend on this progress to suggest future directions, we examined the published literature on MNDs in SSA to identify disease prevalence, genetic and other risk factors. Our findings indicate that the amount of research evidence on MNDs in SSA is scanty. We identified only a few (eight) genetic studies, mostly conducted in South Africa. These studies strongly suggest that there appear to be population-specific causes of MNDs among Africans and also between African and non-African populations. Further studies, especially molecular, genetic and genomic studies among African populations, will be required to help advance our understanding of MND biology. Insights from these studies would help to improve the timeliness and accuracy of clinical diagnosis and treatment.

## 2. Methodology

### 2.1. Data Sources

**Figure 1:**
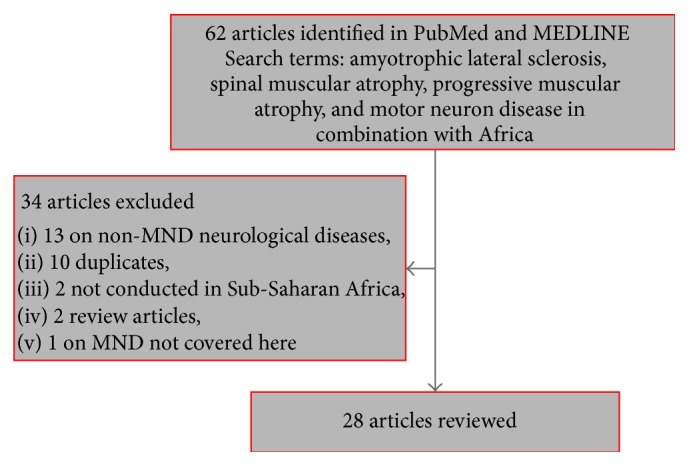
Flow diagram showing the selection of articles for review.

We searched PubMed and MEDLINE via EBSCO for articles investigating MNDs and published up until April 2015. We used a number of search terms, including “amyotrophic lateral sclerosis”, “spinal muscular atrophy”, “progressive muscular atrophy”, and “motor neuron disease” in combination with “Africa” or “sub-Saharan Africa”. We also scanned the references from the articles obtained to identify potentially useful articles that would fit our search criteria. We then evaluated the articles (titles, abstracts, and then full texts) to find out if they met our inclusion criteria (Figure 1). The inclusion criteria were that an article must (i) have reported on MNDs (specifically ALS, SMA, and PMA), (ii) be a primary research article, (iii) have been conducted in SSA, and (iv) have been indexed in the databases up until April 19, 2015.

### 2.2. Article Selection, Data Extraction, and Assessment

Articles meeting all four conditions indicated above were selected for further analysis. Studies reporting on other forms of MNDs (such as Konzo), other neurological disorders (including Parkinson's disease and Alzheimer's disease), and HIV-related neurocognitive disorders were excluded. No restrictions were made in terms of study design but all duplicate items, studies conducted outside SSA, and review articles were excluded. Figure 1 summarises the article selection process.

## 3. Results and Discussion

Twenty-eight articles that matched our search criteria were selected for further analysis. Details of these studies have been provided in Table 1.

### 3.1. MND Epidemiology and Risk Factors in SSA

Twenty-eight studies were reviewed (13 retrospective, 8 case-controlled, 2 cross-sectional, and 5 case studies). Classifying articles based on study settings, we obtained twenty-five hospital-based and two community-based studies as well as one systematic study on ALS, SMA, and PMA (Table 1). These studies were conducted in a dozen countries in SSA, including South Africa (5 studies), Nigeria (5 studies), Senegal (4 studies), Mali (3 studies), Ethiopia (2 studies), multiple countries in Sub-Saharan Africa (2 studies), and Ivory Coast, Tanzania, Congo, Zimbabwe, Cameroon, Sudan, and Kenya (one study each; Table 1). The number of study participants with MNDs ranged from 2 to 116. The two community-based studies estimated disease prevalence rates to be 5/100,000 and 15/100,000 in Ethiopia and Nigeria, respectively (Table 1). Three of the hospital-based retrospective studies gave the prevalence rates as 750/100,000, 290/100,000, and 250/100,000 in Ivory Coast, Senegal, and Cameroon, respectively. The diagnostic criteria and methods used varied across studies, but electromyography was carried out in seven studies. The proportion of males involved in these studies was often higher than females. In addition, the age of MND patients in all the studies reviewed ranged from 12 to 84 years.

SMA and ALS were reported in 10 and 11 articles, respectively. A study conducted in Nigeria [18] reported on all three MNDs under consideration (ALS, SMA, and PMA). In this report, 73 patients who were suffering from ALS, as well as 9 and 10 suffering from SMA and PMA, respectively, were studied [18]. SMA type 1 was also reported in a few infants.

Furthermore, seven studies investigated risk factors for MNDs. The risk factors reported included severe hypotonia in infants, trauma, family history of MNDs, sensory changes, and spinal anaesthesia. With regard to this, two studies [11, 28] reported on family history and severe hypotonia in infancy as possible risk factors for SMA type 1, while Abdulla et al. [31] also observed family history of MNDs to be a risk factor for 14% of patients. Imam and Ogunniyi [9] identified trauma in 37.5% of ALS subjects while Wall and Gelfand [24] observed sensory changes in six participants. Additionally, while Bauer et al. [27] found spinal anaesthesia to be a risk factor for muscular atrophy in 7 out of 117 spinal anaesthesia inpatient admissions, Lekoubou et al. [30] reported that diabetes mellitus may not have any causative association with ALS.

### 3.2. Molecular and Genetic Studies into MNDs in SSA

Eight studies were focused on identifying genetic causes of MNDs. In 1988, Kiepiela and colleagues [20] evaluated the physiology of immunoregulatory cells in SMA among African patients and healthy controls. The study did not identify any cellular differences between SMA patients and control subjects. In 1999, Stevens et al. [14] identified homozygous deletions in the Neuronal Apoptosis Inhibitory Protein (*NAIP*) gene among 14% of 29 SMA patients studied. Homozygous* SMN1* deletions were also found in 65.5% (19/29) of black patients studied (compared to the same genetic deletions identified among 90–95% of patients of white ancestry). It was also observed that 47% of these* SMN1* deletions were in telomeric exon 7 but not exon 8. No such genetic deletions were identified in the remaining 35% (10/29) of black patients studied [14]. Following haplotype analysis of the genomes of these patients without genetic deletions (using six closely linked markers), no evidence for a founder mutation was observed [14]. This was contrary to later findings from an investigation on Finnish patients, which identified that the families of the patients sampled shared a common haplotype, indicating the possibility of a disease-related common founder mutation among this study population [39]. Based on these findings, it was suggested that the molecular basis of SMA onset and progression might differ between South African patients of different racial ancestries [14].

Wilmshurst et al. [15] explored this further through genetic studies in thirty unrelated and racially diverse SMA patients (including 12 black patients) in the Western Cape area of South Africa. In this retrospective study, four of the patients recruited were suffering from SMA type 1, sixteen presented with SMA type 2, and ten were suffering from SMA type 3. Contrary to the earlier report [14], Wilmshurst and colleagues [15] reported 100% homozygous deletions of exon 7 or exons 7 and 8 of the* SMN1* gene in all patients and concluded that the study subjects were not genetically or phenotypically different from internationally recognised forms of SMA. The differences in the outcomes of the two studies, though, could be due to the fact that the latter study excluded patients who showed facial musculature. Such patients were, however, included in the earlier study. In a later investigation that included patients showing facial musculature, less homozygous deletions of the* SMN1* gene (51% deletions, compared to the 100% earlier reported) in black South African patients were observed [13]. These findings were similar to those of Stevens and colleagues that identified genetic deletions in 65.5% of patients [14]. Labrum et al. [13] also reported SMA to be less common in black populations than white ones. Carrier rates of 1/50 and 1/23 and predicted birth rates of 1/3574 and 1/1945 in South African black and white populations, respectively, were provided. Apart from extreme hypotonia from infancy observed in an SMA type 1 patient in Kinshasa, Congo, a family history of similar clinical symptomatology in the patient's elder brother was also recorded [11].* SMN1* deletions were confirmed in the primary patient's genome following a quantitative polymerase chain reaction-based testing. In addition, two normal* SMN2* alleles were found in the patient's genome [11].

Upon investigating two sisters in Mali with spastic paraplegia and marked atrophy of the distal upper extremities, another study reported an extended homozygosity at chromosome 19p13.11-q12 (designated as SPG43) shared by patients but not shared by controls (controls consisted of 5 extended family members and 43 unrelated individuals of the same ethnic group) [34]. SPG43, they reasoned, could be a genetically distinct form of hereditary amyotrophy with spastic paraplegia. Following up on this outcome, it was observed in a later investigation that the (c.187G>C; p.Ala63Pro) homozygous missense variant in* C19orf12* existed in patients who were suffering from spastic paraplegia with amyotrophy [38]. However, this mutation was not found in the 298 Malian controls [38]. Interestingly, upon screening several alleles in the National Heart, Lung, and Blood Institute exome sequencing project database, this genetic mutation was identified in three out of 3836 alleles of African Americans screened but none in the 8222 European-American alleles [38], suggesting that genetic risks for this disease are likely to be ethnic-dependent. This sustains the need for major MND genetic studies in Africa. Finally, SMN copy numbers and SMA carrier frequencies were also investigated in 628 Malians [17]. The SMA carrier frequency was estimated to be 1/209, much lower than the frequency that had been previously reported for Eurasians (1/30–50) [17]. It was further suggested that Malians and other Sub-Saharan Africans were more likely to have three or more copies of* SMN1* (a higher copy number than Eurasians) and more likely to lack the* SMN2* gene compared to Europeans. This suggests that SMA genetics among natives of SSA might vary from other populations.

It is worth noting that four out of the eight studies that focused on identifying the genetic basis of MNDs investigated South African patients; hence, their findings might not necessarily be applicable to people from other African countries, especially due to recent findings that African populations show some genomic diversity [40]. Aside from the four genetic studies conducted in South Africa, three more reports were on research done in Mali, with the remainder focusing on a patient in Kinshasa, Congo. Moreover, most of the studies (six of them) examined risk factors for SMA much to the neglect of PMA and ALS (there were only two studies examining amyotrophy together with spastic paraplegia). Altogether, the available data on genetic and molecular basis of MNDs among African populations is lacking, with no information available for populations in most countries. Due to this, we emphasise the need for further characterisation of genes and noncoding elements to further our understanding of MND disease mechanisms and propagation among African populations.

### 3.3. The Need for Further Studies

With some studies suggesting much less homozygous deletions of the* SMN1* gene in black South African SMA patients as compared to patients from other continents [13, 14], there is an increasing need to fully characterise the genetic basis of SMA and other MNDs among Africans and also to identify possible differences between Africans and other populations. In this section, we discuss how further genetic studies into the basis of MNDs in African populations would enhance our understanding of MNDs and support neurological research and medical care in Africa. We also highlight some recent neuroscience research-promoting initiatives that scientists in Africa can take advantage of in order to help advance their research expertise and output.

#### 3.3.1. Advancing Drug Development, Clinical Diagnosis, and Care

The clinical picture of MNDs in black Africans was first noted in 1990; disease characteristics differed slightly from what had previously been observed in white patients [13, 36]. It was reported in a study of forty-five African children with SMA that the clinical manifestation of the disease differed from what had been observed worldwide in two respects [36]. The first reason was the paucity in family history of only 9% in the black children studied as compared to 70% in other populations earlier identified by Smith and Patel in 1965 [36]. The second reason was the frequent involvement of facial muscle dysfunction, with 80% of SMA type 1 patients showing “expressionless faces.” The facial musculature symptoms initially led to a diagnosis of congenital myopathy, which was later challenged by the results of a muscle biopsy [13, 36]. The identification of genetic elements underlying these ethnic-dependent phenotypic variations is a strong suggestion that the mechanisms and propagation of MNDs might be population-specific, meaning that one-size-fits-all drugs and diagnosis platforms would not be effective for all patients. Further neurogenomic and neurogenetic studies might provide significant findings that would help to improve the clinical diagnosis and care of MNDs, as well as accelerate the development of better-targeted therapies [41].

#### 3.3.2. MND-Based Biomedical Research

The lack of MND research in most parts of Africa can be partly blamed on the lack of adequately trained and equipped scientists conducting neuroscience research on the continent [42, 43]. However, it is rather fair to mention that the lack of world-class research facilities and neuroscience training programmes in several African countries discourages young researchers and students from taking up neuroscience as a carrier choice, leading to the glaring disparity in neuroscience research capacity and output between Africa and more-developed continents [42–45]. Moreover, the difficulty in obtaining research funding, especially funds awarded by governments and national funding schemes, has been a major hindrance to quality research [42, 43].

Nevertheless, the situation seems to be improving in recent times, with the introduction of specific training programmes aimed at helping to build sustainable capacity for neuroscience research and education in Africa. New training programmes such as short courses, workshops, and degree programmes have been introduced for scientists involved in neuroscience research in Africa, to help improve the capacity of these scientists to conduct more high-impact research. These include workshops organised by institutions like TReND (Teaching and Research in Natural Sciences for Development in Africa) (http://www.trendinafrica.org/) and IBRO (International Brain Research Organization) to train African scientists on how best to use neurogenetics tools and model organisms (such as fruit fly,* Drosophila melanogaster*) which are powerful for molecular and genetic aspects of neuroscience [42, 43]. The cost-effectiveness of such tools and organisms also makes them suitable for areas where research funding is scarce, as the case is in most countries in Africa [43]. Institutions like Adéquation (http://adequationgermany.embl.de/), Seeding Labs (http://seedinglabs.org), Korle Bu Neuroscience Foundation (http://kbnf.org), and TReND have also contributed immensely by partnering with African universities, companies, and hospitals to provide functional laboratory and medical equipment to support local neuroscience education and research [42, 43]. Detailed discussions about these support systems led by nonprofit organisations have been previously provided [41–43, 46]. To help improve the use of genomics tools and techniques in health-related research in Africa, the H3Africa (Human Heredity and Health in Africa, http://h3africa.org) initiative is making great strides by supporting African scientists with dedicated research funding, training, and intracontinental collaboration opportunities [46–48]. This provides opportunities for more scientists working in neuroscience-related areas to apply genomics and genetics research to help address research questions [41]. However, for the long-term sustainability of these initiatives, there is the need for the development of more top notch, interdisciplinary undergraduate and postgraduate programmes in neuroscience in African universities. This would ensure that more African scientists, clinicians, and students are trained to carry out research in neuroscience, in order to help provide further insights that would contribute to improving neurological healthcare, social care, and public perceptions about neurological diseases [43].

Biomedical research in Africa currently depends heavily on donor support from developmental partners; however, the research priorities tied to these support systems might not necessarily be aligned with local research priorities in the respective African countries [46, 49, 50]. Moreover, the highly competitive nature of these funding schemes (since they are often open to many countries in the developing world) reduces the chances of African early-career scientists and senior scientists with low academic achievements from coming out successful [46]. To improve excellence in neuroscience research in Africa (particularly those focusing on MNDs), African governments and other local agencies must improve their investments in funding the provision of world-class resources and training programmes in this area. Specifically, there is the need for governmental funding support at the national, regional, and local government levels. For this reason, leaders of the African Union endorsed a target for member states to spend at least 1% of their gross domestic product on local research and development [47]. However, about a decade later, little has changed in terms of national research funding. Research in Africa is still almost fully supported by international funding agencies. In fact, the African Innovation Outlook 2010, a survey of scientific productivity in the different African countries, showed that only three countries (South Africa, Malawi, and Uganda) topped the 1% spending threshold in 2007, with most countries remaining far from the mark [47]. This demonstrates that there is a big research-funding gap in Africa, which needs to be filled. Aside from governmental support, contributions from industries, charity organisations, and philanthropists would also help to increase the quality of neuroscience research conducted in Africa. On the other hand, more scientists should get involved in public engagement activities in order to help policymakers and the public better understand the importance of scientific research and the need to increase investments in this area.

## 4. Conclusion

In this study, we have summarised the body of literature on ALS, SMA, and PMA in SSA. We found that the amount of research conducted in this area is limited, with most studies conducted in only a few countries. Due to this, we emphasise the need for more and larger-scale molecular, genetic, and genomic studies into these MNDs among different African populations. The previous discovery that some genetic underpinnings of MNDs such as homozygous* SMN1* deletion frequency and genetic mutations in SMA vary between Eurasians and specific populations in SSA is one of the important reasons supporting the need for further studies to identify currently unknown factors regulating MNDs among African populations. Conducting further research into the molecular and genetic bases of MNDs could potentially lead to the identification of clinically relevant biomarkers for disease diagnosis, management, and treatment. In the long term, this would help to improve the timeliness and accuracy of clinical diagnosis and treatment, as well as social care and public perception about MNDs.

## Figures and Tables

**Table 1:** Overview of studies into MNDs in Sub-Saharan Africa^*∗*^.

Article reference	Country and setting	Study design and year	Sample characteristics	Diagnostic criteria and tools used	Disease prevalence	Disease risk factors and genetic determinants identified
Wilmshurst et al. [15]	South Africa; hospital-based	Case-controlled studies; 1980–2001	30 patients: 4 SMA type 1 patients; 16 SMA type 2 patients; 10 SMA type 3 patients. All patients were black South Africans. Six horn cell disease patients were used as controls	Clinical diagnosis	NA	Homozygous deletions of exon 7 or exons 7 and 8 of the *SMN1* gene in all patients. Patients were found not to be genetically or phenotypically different from internationally recognised forms of SMA

Ekenze et al. [16]	Nigeria; hospital-based	Retrospective; 2003–2007	8440 patients: 1249 patients (640 men) suffering from neurological disorders (45 y mean age). Ten of these patients were suffering from ALS (4 were men)	NA	800/100,000	NA

Sangaré et al. [17]	Mali; hospital-/laboratory-based	Case-controlled studies	628 Malians, 120 Nigerians, and 120 Kenyans (healthy individuals)	NA	1/209 SMA carrier frequency in the SSA patients (mostly Malians) compared to 1/30–50 in Europeans and Asians	Participants had 3 or more copy numbers of *SMN1* gene and lacked *SMN2* compared to Europeans

Osuntokun et al. [18]	Nigeria; hospital-based	Retrospective; 1958–1973	92 patients with MND: 73 ALS, 9 SMA, and 10 PMA	ENMG, muscle biopsy	21/100,000	NA

Stevens et al. [14]	South Africa; hospital-based	Case study series	29 SMA patients	Clinical	NA; the study concluded that differences in SMA phenotype in black patients may be due to different molecular/genetic basis mediating the disease among such populations	65.5% (19/29) homozygous *SMN1* deletions in black patients. 47% (4/29) of *SMN1* deletions were found in telomeric exon 7 but not exon 8. Also, 14% homozygous *NAIP* deletions were identified. No gene deletions were found in 35% of patients

Adam [19]	Kenya; hospital-based	Retrospective; 1978–1988	47 MND participants (35 men and 12 women). 18 of these were suffering from ALS (13–80 y olds)	Clinical, 1/3 ENMG	NA	NA

Kiepiela et al. [20]	South Africa; hospital-/laboratory-based	Case-controlled studies	South African and Indian SMA patients	Clinical	NA	Genetics: no significant changes in patients' immunoregulatory cells identified

Kengne et al. [21]	Cameroon; hospital-based	Retrospective; 1993–2001	4041 patients; 145 with neurological diseases. Ten out of these were suffering from ALS (8 men and 2 women); mean age = 50.9 y	NA	250/100,000 of all neurologic consultations; 12% of all neurological cases	NA

Imam and Ogunniyi [9]	Nigeria; hospital-based	Retrospective; 1980–1999	16 ALS patients (15 men and 1 woman), 16–60 y	El Escorial diagnostic criteria	NA	Identified risk factor: trauma in 37.5% of subjects

Sene et al. [22]	Senegal; hospital-based	Retrospective; 1999-2000	33 ALS patients	El Escorial diagnostic criteria, ENMG	NA	NA

Tekle-Haimanot et al. [23]	Ethiopia; community-based	Cross-sectional; 1986–1988	60820 participants (29412 men), 59% < 20 y, 3 MND patients (2 men and 1 woman)	Neurological examinations, screening questionnaire	5/100,000	NA

Labrum et al. [13]	South Africa; hospital-based	Case-controlled studies	116 SMA patients; 92 of black ancestry, 24 white patients	Clinical (not ENMG)	Carrier frequency of 1/50 in black population but 1/23 in white population	51% homozygous deletions of *SMN1* gene in black patients, as opposed to 95% of patients worldwide

Wall and Gelfand [24]	Zimbabwe; hospital-based	Retrospective; 1967–1971	13 MND patients; 24–55 y	Clinical (not ENMG)	NA	Sensory changes in six participants

Lumaka et al. [11]	Kinshasha, Congo; hospital-based	Case study	1 SMA type 1 infant patient	Clinical, ENMG	NA; family history (similar symptomatology in elder brother)	Extreme hypotonia in infancy; homozygous deletions of *SMN1*; 2 *SMN2* alleles present

Collomb et al. [25]	Senegal; hospital-based	Retrospective; 1960–1968	18 ALS patients (17 men and 1 woman), 25–70 y	Clinical (not ENMG)	NA	NA

Pelleboer et al. [26]	Nigeria; hospital-based	Case study	1 SMA type 1 infant	Clinical	NA	NA

Bauer et al. [27]	Tanzania, hospital-based	Case-controlled series; 1993–1997	117 SA inpatient admissions and 117 matched controls; 24–77 y	Neurological examination, screening questionnaire	7/117 in SA group but 0/117 in control group	SA was identified as a risk factor for muscular atrophy

Ndiaye et al. [28]	Senegal; hospital-based	Case study series	5 SMA type 1 patients	Clinical, ENMG	NA	Identified risk factors: severe hypotonia in infancy, family history of SMA

Osuntokun et al. [29]	Nigeria; community-based	Cross-sectional	18954 participants; 9282 men, 58% < 20 y, 11% > 50 y	Screening questionnaire	MND; 15/100,000	NA

Lekoubou et al. [30]	SSA; systematic analysis of associations between MNDs and diabetes mellitus	Retrospective (3 case control and 2 cross-sectional studies)	Up to 2371 ALS cases reviewed	NA	NA	No association between ALS and diabetes mellitus identified

Abdulla et al. [31]	Sudan; hospital-based	Retrospective; 1993–1995	28 MND patients, including 19 ALS patients	Clinical, ENMG	NA	Family history of MND identified in 14% of patients

Cosnett et al. [32]	South Africa; hospital-based	Retrospective; reviewed 9.5 y of cases	59 blacks (47.4 y mean age), 9 Indians, 16 whites, 2 coloured patients (54 y mean age for non-black patients)	Clinical, 45% ENMG	The following prevalence values per 100,000 persons were observed for different populations: blacks (0.88), whites (2), coloured (7), Indians (1.4).	NA

Harries [33]	Ethiopia; hospital-based	Case study series; 1954	2 male participants, aged 26 and 30 y	Clinical (no ENMG)	NA	NA

Meilleur et al. [34]	Mali; hospital/laboratory-based	Case control	Study cohort included 2 spastic paraplegia patients with amyotrophy, 5 extended family members, and 43 unrelated people of the same ethnic group	Clinical	NA	Extended homozygosity at chromosome 19p13.11-q12 (designated as SPG43) in patients but not controls

Jacquin-Cotton et al. [35]	Senegal; hospital-based	Retrospective; 1960–1969	6100 patients; 18 ALS (16 men), 25–70 y	Clinical (no ENMG)	290/100,000	NA

Moosa and Dawood [36]	SSA	Case study series	45 SMA patients; 15 SMA type 1, 19 type 2, and 9 type 3; infants to 48 month olds; 1 : 1.7 female/male ratio	Clinical, ENMG	NA	Facial weakness in 80% of type 1 patients

Piquemal et al. [37]	Ivory Coast; hospital-based	Retrospective; 1971–1980	4000 patients; 30 ALS (22 men), 50% < 40 y	Clinical (no ENMG).	750/100,000	NA

Landouré et al. [38]	Mali; hospital- and laboratory-based	Case control	Study cohort included 2 spastic paraplegia patients with amyotrophy, 298 Malian controls, and several alleles found in the NHLBI exome sequencing Project database	Clinical	NA	Homozygous missense variation of c.187G>C; p.Ala63Pro, in *C19orf12*, was observed in the spastic paraplegia patients but not in the 298 Malian control subjects

^*∗*^ALS, amyotrophic lateral sclerosis; ENMG, electroneuromyography; MND, motor neuron disease; NA, not available; PMA, progressive muscular atrophy; SMA, spinal muscular atrophy; y, years; m, months; SA, spinal anaesthesia; *SMN1*, Survival Motor Neuron 1 gene; *NAIP*, Neuronal Apoptosis Inhibitory Protein; NHLBI, National Heart, Lung, and Blood Institute.

## Abbreviations

ALS: Amyotrophic lateral sclerosis
MNDs: Motor neuron diseases
SMA: Spinal muscular atrophy
SSA: Sub-Saharan Africa
ENMG: Electroneuromyography
SA: Spinal anaesthesia
*SMN1*: Survival Motor Neuron 1 gene
*NAIP*: Neuronal Apoptosis Inhibitory Protein
*SOD1*: Superoxide dismutase
SPG43: Chromosome 19p13.11-q12
NHLBI: National Heart, Lung, and Blood Institute.
